# A highly sensitive, PCR-based method for the detection of *Plasmodium falciparum *clones in microtiter plates

**DOI:** 10.1186/1475-2875-7-222

**Published:** 2008-10-29

**Authors:** Steven P Maher, Bharath Balu, Doug A Shoue, Matthew E Weissenbach, John H Adams

**Affiliations:** 1 Department of Global Health, College of Public Health, University of South Florida, Tampa, Florida, USA; 2 University of Notre Dame, Notre Dame, Indiana, USA

## Abstract

**Background:**

Cloning of parasites by limiting dilution is an essential and rate-limiting step in many aspects of malaria research including genomic and genetic manipulation studies. The standard Giemsa-stained blood smears to detect parasites is time-consuming, whereas the more sensitive parasite lactate dehydrogenase assay involves multiple steps and requires fresh reagents. A simple PCR-based method was therefore tested for parasite detection that can be adapted to high throughput studies.

**Methods:**

Approximately 1 μL of packed erythrocytes from each well of a microtiter cloning plate was directly used as template DNA for a PCR reaction with primers for the parasite *18s rRNA *gene. Positive wells containing parasites were identified after rapid separation of PCR products by gel electrophoresis.

**Results:**

The PCR-based method can consistently detect a parasitaemia as low as 0.0005%, which is equivalent to 30 parasite genomes in a single well of a 96-well plate. Parasite clones were easily detected from cloning plates using this method and a comparison of PCR results with Giemsa-stained blood smears showed that PCR not only detected all the positive wells identified in smears, but also detected wells not identified otherwise, thereby confirming its sensitivity.

**Conclusion:**

The PCR-based method reported here is a simple, sensitive and efficient method for detecting parasite clones in culture. This method requires very little manual labor and can be completely automated for high throughput studies. The method is sensitive enough to detect parasites a week before they can be seen in Giemsa smears and is highly effective in identifying slow growing parasite clones.

## Background

Isolating clones of *Plasmodium falciparum *is a prerequisite to several different areas of malaria research. For example, many genomic and genetic manipulation studies require single clones of the parasite for valid and accurate analyses [1,2]. Experimental analysis of field samples also typically requires clonal selection of parasites from mixed infections to accurately associate phenotype characteristics with specific genotypes [3].

Cloning is routinely accomplished by diluting the parasite culture with uninfected erythrocytes into a 96-well microtiter plate at 0.5 or 0.25 parasites per well. After three weeks of growth, wells containing parasites usually can be detected in a Giemsa-stained blood smear or by using a parasite lactate dehydrogenase (pLDH) assay [4-6]. Most basic clinical and research laboratories are adequately equipped to prepare and examine Giemsa-stained smears, although it is a time-consuming procedure with limited scalability for simultaneous analysis of multiple cloning plates. While the pLDH assay can process several plates simultaneously, it involves multiple steps and requires several freshly made reagents. Here, a novel PCR-based method for parasite clone detection is described that is simple, highly sensitive and easily adaptable to high-throughput studies in most laboratories.

## Methods

### Detection of parasites by PCR and agarose gel electrophoresis

For parasite detection, PCR was performed with primers for the parasite *18s rRNA *gene (PlasmoDB [7] ID: MAL1_18s; forward primer: 5'-AACCTGGTTGATCTTGCCA-3'; reverse primer: 5'-GTATTGTTATTTCTTGTCACTACCTCTC-3'). A PCR master mix was first created by adding 1 mL of GoTaq 2× Master Mix (Promega Inc.), 720 μL of nuclease free water and 200 nM of each primer. Eighteen μL of master mix was transferred to each well of a 96 well PCR plate (Fisher scientific) and a 2 μL scrape of parasitized erythrocytes was directly transferred, using a multichannel pipet, from the bottom of each well of the culture plate to serve as templates. PCR was performed using Eppendorf Mastercyclers under the following conditions: 94C for 2 min followed by 35 cycles of 94C for 10s, 50C for 30s and 65C for 1 min.

Ten μL PCR product was then directly loaded (as the GoTaq master mix contains a DNA loading dye), via a multichannel pipet, into a 2% agarose gel and separated at 150 V for approximately 15 minutes. The agarose gels were stained with ethidium bromide and images recorded using a gel documentation system (Bio-Rad).

### Estimating the parasite detection limit of PCR

Mature blood stage parasites were first purified from a *P. falciparum *NF54 culture using a magnetic column (Miltenyi Biotech) as described before [2]. The purified parasites were counted using a haemocytometer and 304,716 parasites were added to 1.6 mL of culture media and 80 μL of 50% haematocrit to yield a culture master mix with 2.5% haematocrit and 0.15% parasitaemia. Two hundred μL of the master mix was then added to each of the eight wells in a column of a 96-well microtiter plate yielding approximately 38,000 parasites per well. One hundred μL of culture from the first row of the plate was then serially diluted 1:1 across the plate by mixing with 100 μL of culture blood with 2.5% haematocrit. The plate was then centrifuged at 1250 g for 3 min to pellet the erythrocytes and a 1 μL scrape was taken from the bottom of the plate and used as template in the PCR. The 1 μL scrape contained approximately 0.5 μL of packed blood that equalled 1/5^th ^of the total erythrocytes in each well. Therefore, the number of parasites added to the PCR reaction was calculated accordingly across the plate.

### Detection of parasite clones by PCR

Limiting dilutions were carried out on two genetically manipulated *P. falciparum *NF54 cultures by diluting parasites at 0.5 parasites/well with uninfected erythrocytes as described before. Two 96-well cloning plates were initiated at 2.5% haematocrit in 200 uL of RPMI 1640 medium containing 0.5% Albumax II, 0.25% sodium bicarbonate and 0.01 mg/ml gentamicin. The culture media was changed twice a week and 0.5% of fresh haematocrit was added once a week and parasite cultures were maintained at 37°C in three-gas incubators at 5% CO_2_, 5% O_2 _and balance N_2 _until detection.

On days 10, 12, 14 and 16, PCR was performed on each plate by using a 2 μL scrape from the bottom of every well and positive clones were detected after gel electrophoresis. On days 17 and 24, every well from the two plates was smeared, fixed, stained by 20% Giemsa stain (Sigma) and scanned by light microscopy (Nikon Labophot 2, 100×).

## Results

### Parasite detection limit of PCR

**Figure 1:**
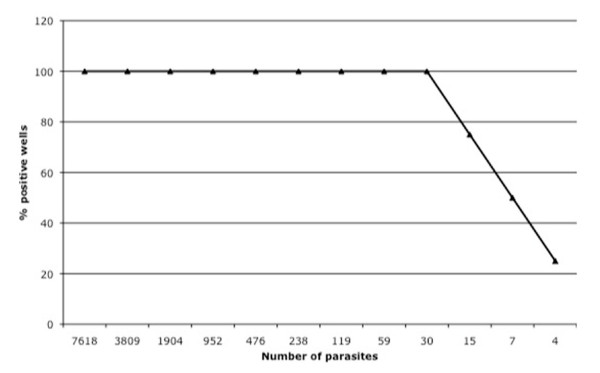
**Parasite detection limit of PCR**. Number of parasites used in the PCR reaction plotted against the percentage of times they were detected positive from eight wells revealed that as few as 30 parasites (from a well with 0.0005% parasitaemia) can be detected consistently using this method.

To estimate the number of parasites needed for detection by PCR, mature blood-stage parasites were purified by using a magnetic column and added to one column (eight wells) of a 96-well plate to obtain a parasitaemia of 0.15% at 2.5% haematocrit. The parasite culture was then serially diluted 1:1 to obtain parasitaemias ranging from 0.15% to 0.00007%. A PCR using 0.5 μL of packed erythrocytes on the entire plate detected as low as 0.00007% parasitaemia (approximately four parasites/reaction) in some wells and parasitaemias of 0.0005% (approximately 30 parasites/reaction) and above were consistently detected in all wells (Figure 1).

### Detection of parasite clones by PCR

**Figure 2:**
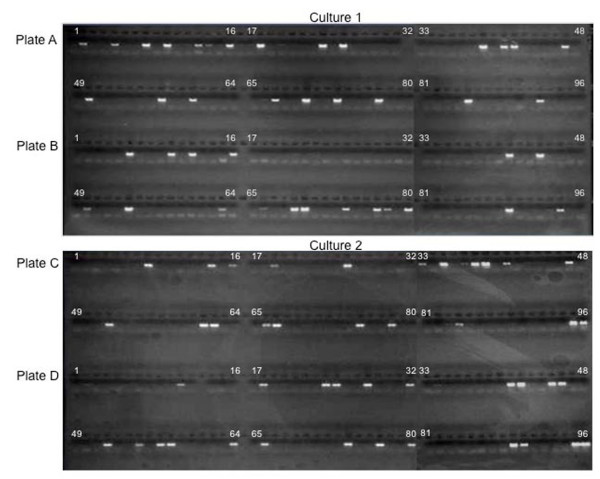
**Detection of parasites in cloning plates**. PCR reactions using primers for the parasite *18s rRNA *gene were performed on samples from the individual wells of four cloning plates from two different *P. falciparum *cultures on day 12, post-dilution. Agarose gel electrophoresis revealed a 500 bp, parasite-specific fragment, efficiently amplified from several positive wells in all four cloning plates that were confirmed by Giemsa-stained blood smears.

Having confirmed the feasibility and efficiency of direct parasite detection by PCR, the technique was further tested to detect parasites in actual cloning plates. Limiting dilution was performed on two genetically manipulated *P. falciparum *NF54 culture into two 96-well microtiter plates each. PCR analysis on the four cloning plates detected parasites on days 10, 12, 14 and 16 post-dilution (Figure 2). Even though parasites were detected in some wells by day 10, additional positive wells were found on day 12, but none after that (Table 1). On day 17, all four plates were checked by Giemsa-stained blood smears and parasites were seen in all the wells that were determined to be positive by PCR. However, the Giemsa smears did not identify parasites in 18 out of 87 clones that were determined to be positive by the PCR method. A second Giemsa smear made on day 24 verified the presence of parasites in these 18 wells, thus confirming early detection of parasites by PCR much before they can be seen in Giemsa smears.

**Table 1:** Comparison between Giemsa stain and PCR methods for detecting parasite clones in four 96-well microtiter plates.

Day post-dilution	Number of clones identified by	
	Giemsa	PCR
10	0	34
12	0	87
14	44	87
16	58	87
17	69	
24	87	

## Discussion

Cloning of *P. falciparum *by limiting dilution is a technique routinely used in malaria research labs. Current methods available for clone detection are labour intensive and time consuming. The successful development of a PCR-based method for detecting parasite clones offers several advantages over the current detection methods: (1) the entire procedure to screen 400 individual cultures can be performed in < 2 hours by one person; (2) the method is highly sensitive and can detect cloned parasites in < 2 weeks; and (3) for higher throughput studies the protocol can be fully automated via robotics.

Other than the obvious incentives in using this protocol due to the increased efficiency of the screening procedure, the shortening of time and effort required to identify the positive clones also creates significant cost savings by significantly shortening the initial stages of experiments. These savings will be enhanced in studies involving genetically manipulated parasites (e.g., gene knockout) where a slow-growing phenotype may require much longer time to reach a detectable threshold. Moreover, in such cases, it might be harder to detect parasites using the currently available methods even after three weeks of growth, after which there is considerable lysis of erythrocytes, resulting in the loss of the culture. Such slow growing clones can be identified sooner by using the PCR method and transferred to higher volume cultures before culture lysis.

This method can also be exploited in scenarios with limited parasite material, such as analysis of field isolates, as it circumvents the need to isolate the parasite genomic DNA and instead allows direct use of infected blood.

## Conclusion

The PCR-based method is a simple, effective, and efficient technique to detect parasite clones with minimal manual labour. As malaria research progresses into the future, whole-genome approaches involving sophisticated robotics are becoming more commonplace, and this method compliments such sophistication.

## Competing interests

The authors declare that they have no competing interests.

## Authors' contributions

BB, DAS and JA conceived of the study. SM and BB designed the experiments. SM, BB, DAS and MEW carried out the experiments. SM, BB and JA drafted the manuscript.
